# Do polyphenol-rich solutions derived from tropical fruit seeds protect dentin against erosive–abrasive challenges?

**DOI:** 10.1007/s00784-026-06961-1

**Published:** 2026-06-20

**Authors:** Eveline Lassance Cunha de Alencar Camacho, Paula Mendes Acatauassu Carneiro, Sandro Cordeiro Loretto, Roberta Pimentel de Oliveira, Aryvelto Miranda Silva, João Felipe Besegato, Wilfredo Gustavo Escalante Otárola, Cristiane de Melo Alencar

**Affiliations:** 1 School of Dentistry, State University Center of Pará (CESUPA), Belém, Pará Brazil; 2 School of Dentistry, Federal University of Piauí, UFPI, Teresina, Piauí Brazil; 3 School of Dentistry, Federal University of Mato Grosso do Sul, UFMS, Campo Grande, Mato Grosso do Sul Brazil; 4 School of Dentistry, Catholic University of Santa María, UCSM, Arequipa, Peru

**Keywords:** Dentin erosion, Polyphenolic extracts, Antioxidant activity, Biomodification

## Abstract

**Objectives:**

To evaluate the effect of polyphenol-rich hydroalcoholic extracts derived from the seeds of four tropical fruits—*Paullinia cupana* (guaraná), *Euterpe oleracea* (açaí), *Byrsonima crassifolia* (murici), and *Bactris gasipaes* (peach palm)—on dentin under erosive–abrasive conditions.

**Materials and methods:**

Seed-derived hydroalcoholic extracts were prepared and chemically characterized in terms of total polyphenol content and antioxidant capacity using DPPH and ABTS assays. Human dentin specimens (*n* = 180) were subjected to an initial erosive challenge and randomly allocated into six experimental groups (*n* = 30). Treatments were applied prior to a 7-day erosive–abrasive cycling model. Dentin surface loss (dSL), collagen degradation (dCol), and calcium release (CaR) were quantitatively assessed, and surface morphology was qualitatively evaluated using scanning electron microscopy (SEM). Data were analyzed using one-way ANOVA followed by Tukey’s test (α = 0.05). Exploratory linear regression analyses were performed to assess the association between total polyphenol content and dentin outcomes.

**Results:**

*P. cupana* and *E. oleracea* exhibited the highest polyphenol content and antioxidant capacity and were associated with significantly reduced dSL, dCol, and CaR compared with the untreated control (*p* < 0.05). Qualitative analysis suggested a more compact surface with reduced dentinal tubule exposure in these groups. *B. crassifolia* demonstrated moderate protective effects, whereas *B. gasipaes* showed limited efficacy. Exploratory regression analyses indicated a negative association between total polyphenol content and dentin outcomes.

**Conclusions:**

Polyphenol-rich extracts derived from tropical fruit seeds—particularly *P. cupana* and *E. oleracea*—were associated with protective effects on dentin under erosive–abrasive conditions, reducing mineral loss and collagen degradation. However, the underlying mechanisms remain unclear and should be further investigated in future studies.

**Clinical significance:**

Plant-derived polyphenol-rich extracts may represent a promising adjunctive approach in preventive strategies for erosive tooth wear.

## Introduction

Dental erosion is a progressive and irreversible loss of mineralized tissue caused by chemical dissolution in the absence of bacterial involvement [1]. Its increasing prevalence has become a major concern in contemporary dentistry, particularly among young adults, due to the widespread consumption of acidic foods and beverages [2, 3]. In dentin, erosive wear leads not only to demineralization of the inorganic phase but also to the exposure and subsequent degradation of the collagen matrix, compromising the structural integrity of the substrate and the performance of adhesive restorative procedures [4, 5].

Several approaches have been proposed to minimize the deleterious effects of erosion, including the use of fluoride-based agents, bioactive calcium phosphates, and polymeric coatings [6]. Although conventional fluorides such as sodium fluoride (NaF) remain the most widely used anti-erosive agents, their efficacy in dentin is limited due to the absence of a stable mineral surface for CaF₂-like layer formation [7]. In contrast, stannous- and titanium-containing fluorides (SnF₂ and TiF₄) have shown superior protective effects, attributed to the precipitation of acid-resistant surface complexes and reduced permeability [8]. However, these formulations often present drawbacks related to stability, pH acidity, or esthetic side effects, motivating the search for biocompatible and sustainable alternatives.

In recent years, natural products rich in polyphenolic compounds have gained considerable attention as promising biomodifiers of dentin, due to their ability to modulate biochemical and structural pathways involved in erosive degradation. Studies have demonstrated that plant-derived molecules such as quercetin, epigallocatechin-3-gallate (EGCG), resveratrol, and other flavonoids can significantly reduce dentin demineralization and stabilize the exposed collagen matrix through complementary mechanisms [9–11]. Enhancing the mechanical properties and enzymatic resistance of demineralized dentin collagen is critical to prevent its structural degradation [12]. Collagen crosslinking increases intermolecular interactions, resulting in a denser and more stable network that preserves the triple-helix structure, reduces mechanical breakdown, and limits enzymatic cleavage [13]. Polyphenols have been highlighted for their dual role as crosslinking agents and antioxidants [14]. They strengthen collagen through intermolecular interactions [15] while reducing degradation markers and scavenging reactive oxygen species (ROS) [16], which are involved in collagen breakdown and enzyme activation. Together, these effects contribute to the preservation of the dentin organic matrix.

Polyphenols also inhibit key proteolytic enzymes involved in dentin breakdown. In vitro evidence shows that inhibitors of matrix metalloproteinases (MMPs) and cathepsins—enzymes responsible for collagen degradation—act synergistically to reduce the progression of eroded dentin lesions, reinforcing the concept that modulation of the enzymatic environment is critical for preventing organic matrix collapse [17]. In addition, flavonoids such as quercetin and EGCG are known to interact with the collagen triple helix through hydrogen bonding and hydrophobic interactions, forming exogenous cross-links that increase tensile strength, reduce enzymatic susceptibility, and enhance the long-term stability of demineralized dentin [10].

Furthermore, recent systematic evidence confirms that plant-derived compounds exert dual protective actions: (1) direct modification and strengthening of the dentin substrate, and (2) modulation of the salivary pellicle, increasing its resistance to acidic dissolution [18]. This aligns with findings showing that antioxidant gels containing resveratrol can significantly enhance pellicle-mediated protection, further supporting their potential clinical relevance [11].

The Amazon biome represents one of the richest sources of bioactive plant metabolites worldwide, yet its potential in dental biomaterials research remains largely unexplored. Seeds of native species such as *Euterpe oleracea* (açaí), *Bactris gasipaes* (peach palm), *Paullinia cupana* (guaraná), and *Byrsonima crassifolia* (murici) are known to contain diverse classes of polyphenols, including flavonoids, anthocyanins, tannins, and phenolic acids, all associated with high antioxidant and enzyme-modulating activity [19, 20]. Unlike isolated compounds, these extracts represent complex phytochemical systems in which multiple bioactive constituents may act synergistically, potentially enhancing their biological effects. Moreover, the use of whole extracts better reflects potential clinical applications and supports the valorization of natural resources, contributing to the development of sustainable bioactive strategies in dentistry.

Therefore, the development of experimental polyphenolic solutions derived from these Amazonian fruits may represent an innovative and sustainable strategy to control erosive wear and preserve dentin integrity. This study aimed to evaluate the effect of fruit-derived antioxidant solutions obtained from *Euterpe oleracea*, *Bactris gasipaes*, *Paullinia cupana*, and *Byrsonima crassifolia* seeds on the organic–inorganic interface of eroded dentin, comparing their performance with a commercial stannous fluoride formulation. The null hypothesis of this study was that the different treatments — including fruit seed–derived extracts, a stannous fluoride formulation, and an untreated control — would not affect dentin surface loss, collagen degradation, or calcium release under erosive–abrasive conditions.

## Materials and methods

### Preparation of fruit-derived polyphenolic solutions

*Euterpe oleracea* (1°59′38.5″ S, 47°31′34.6″ W), *Bactris gasipaes* (1°59′37.4″ S, 47°31′34.0″ W), *Paullinia cupana* (1°59′41.4″ S, 47°31′30.0″ W), and *Byrsonima crassifolia* (1°59′47.2″ S, 47°31′30.0″ W) were collected in April 2023 from different sites within the municipality of Mãe do Rio, Pará State, Brazil. Access to the genetic heritage was registered in the Brazilian National System for the Management of Genetic Heritage and Associated Traditional Knowledge (SisGen) under registration number ACDE09A.

For the experimental procedures, the crude hydroalcoholic extracts (HE) were used without further fractionation. The extracts were reconstituted in an ethanol–water solution (70:30, v/v) to obtain a final concentration of 0.75 mg/mL and applied directly to the dentin surface. The pH of each solution was adjusted to near-neutral values prior to application to minimize the influence of acidity on the experimental outcomes.

Seeds from each fruit species (1000 g) were thoroughly washed under running tap water, rinsed with distilled water, and boiled in distilled water for 5 min to remove surface residues. The cleaned seeds were then dried in a forced-air circulation oven (Orion 520, Fanem^®^, São Paulo, Brazil) at 47 °C for four days. After drying, the seeds were ground into a fine powder using a high-speed industrial blender (KD Eletro^®^, São Paulo, Brazil).

Hydroalcoholic extracts (HE) were obtained by macerating the seed powders in 1000 mL of an ethanol–water solution (70:30, v/v) for five days at room temperature under constant stirring. The extracts were filtered and concentrated under reduced pressure in a rotary evaporator (Büchi^®^, Flawil, Switzerland) at 30 °C. The concentrated material was subsequently freeze-dried in a lyophilizer (Christ^®^, Osterode am Harz, Germany) for 72 h at − 101 °C and 23 mm Hg. The resulting dried extracts (HE; 50 g) were stored at − 20 °C until further use.

For liquid–liquid partitioning, the hydroalcoholic extract (50 g) was resuspended in a methanol–water solution (80:20, v/v) and sequentially fractionated with n-hexane (3 × 200 mL), dichloromethane (3 × 200 mL), and ethyl acetate (3 × 200 mL). Each organic phase was separated, and the remaining aqueous phase was designated as the aqueous fraction (AQF). All fractions—n-hexane fraction (HF), dichloromethane fraction (DCMF), ethyl acetate fraction (EAF), and aqueous fraction (AQF)—were concentrated under reduced pressure at 30–40 °C until complete solvent removal. The dried residues were stored in airtight containers at − 20 °C until analysis [20].

For chemical characterization, hydroalcoholic extracts and their respective fractions (10 mg) were dissolved in 1 mL of HPLC-grade methanol and sonicated in an ultrasonic bath (Unique^®^, São Paulo, Brazil) for 20 min to ensure complete solubilization. Chromatographic separation was carried out at room temperature using an Acquity HSS T3 C18 column (100 mm × 2.1 mm, 1.8 μm; Waters^®^, Milford, USA). The mobile phase consisted of (A) 2.5% acetic acid in water and (B) HPLC-grade acetonitrile. The gradient was programmed from 10% to 85% B over 24 min, followed by re-equilibration to 10% B within 10 min, at a flow rate of 0.3 mL min⁻¹. Mass spectrometric detection was conducted in negative ion mode under the following conditions: capillary voltage, 2500 V; end-plate offset, 2000 V; capillary exit, 110 V; skimmer 1, 20 V; skimmer 2, 10 V; dry gas (N₂) temperature, 325 °C; dry gas flow, 1 L min⁻¹; nebulizer pressure, 60 psi; and mass scan range of m/z 200–800. The system was operated at 25 °C.

### Characterization of fruit-derived polyphenolic solutions

The pH of the formulations was measured using a digital pH meter (W3B, BEL Engineering^®^, Monza, Italy). Prior to analysis, the device was calibrated with standard buffer solutions at pH 4.0 and 7.0 according to the manufacturer’s instructions. Measurements were performed under controlled laboratory conditions at room temperature (approximately 25 °C). The pH values were recorded prior to the application of the experimental solutions.

The total phenolic content (TPC) was quantified using the Folin–Ciocalteu colorimetric method adapted from Waterhouse [21]. Briefly, 100 µL of each extract (in triplicate) was mixed with 7 mL of deionized water and 500 µL of Folin–Ciocalteu reagent. After 1–8 min, 1.5 mL of 20% sodium carbonate was added, and the mixtures were kept in the dark for 2 h. Absorbance was recorded at 765 nm using a UV–Vis spectrophotometer (Agilent Cary 60, USA). A calibration curve was constructed with gallic acid standards (10–500 µg mL⁻¹), and results were expressed as micrograms of gallic acid equivalent per milligram of extract (µg GAE mg⁻¹), reported as mean ± standard deviation based on repeated analytical measurements.

The antioxidant capacity was evaluated based on the scavenging activity against the stable radical 2,2-diphenyl-1-picrylhydrazyl (DPPH), following the procedure described by Rufino et al. [19] with minor modifications. The assays were conducted in Nunc MicroWell 96-well flat-bottom plates (Nunclon Delta-treated, Thermo Fisher Scientific, Waltham, MA, USA).

Hydroalcoholic extracts and their solvent fractions were initially dissolved in methanol (HPLC grade) to obtain a stock solution of 500 µg mL⁻¹. Serial dilutions were then prepared to achieve concentrations ranging from 500 µg mL⁻¹ to 7.81 µg mL⁻¹. Subsequently, 100 µL of each dilution was transferred into the wells, followed by the addition of 40 µL of 0.3 mM DPPH solution in methanol. All measurements were performed in triplicate.

For the blank (negative control), 100 µL of methanol and 40 µL of the DPPH solution were added. Trolox (±)-6-hydroxy-2,5,7,8-tetramethylchromane-2-carboxylic acid was used as a positive control to construct the standard calibration curve, at concentrations ranging from 50 µg mL⁻¹ to 0.78 µg mL⁻¹.

The reaction mixture was incubated in the dark for 30 min at room temperature, after which the absorbance was recorded at 492 nm using a microplate spectrophotometer. The half-maximal inhibitory concentration (IC₅₀) value—representing the concentration required to scavenge 50% of the DPPH radicals—was determined by linear regression analysis.

The antioxidant potential was assessed by measuring the ability of the samples to quench the 2,2′-azino-bis(3-ethylbenzothiazoline-6-sulfonic acid) diammonium salt radical cation (ABTS•⁺), following the procedure described by Rufino et al. [19] with minor modifications.^14^ The method is based on the reduction of the blue–green ABTS•⁺ chromophore to its colorless form upon reaction with hydrogen-donating antioxidants.

Hydroalcoholic extracts and their solvent fractions were dissolved in HPLC-grade methanol to obtain a stock solution of 500 µg mL⁻¹. Serial dilutions were prepared to yield concentrations ranging from 500 µg mL⁻¹ to 7.81 µg mL⁻¹. In each well of a 96-well microplate, 20 µL of each dilution and 280 µL of the freshly prepared ABTS•⁺ solution were added. All reactions were carried out in triplicate.

For the blank (negative control), 20 µL of methanol and 280 µL of the ABTS•⁺ solution were added to the wells. Trolox (±)-6-hydroxy-2,5,7,8-tetramethylchromane-2-carboxylic acid was used as the reference antioxidant to construct the calibration curve, at concentrations ranging from 500 µM to 50 µM. The reaction mixtures were incubated in the dark for 20 min at room temperature, and absorbance was measured at 734 nm using a microplate spectrophotometer.

### Ethical aspects

This study was conducted in accordance with the ethical principles outlined in the Declaration of Helsinki and followed the recommendations of the International Committee of Medical Journal Editors (ICMJE). Human dentin specimens were obtained from extracted third molars donated by volunteers who provided written informed consent. Human saliva was also collected from healthy adult donors under informed consent. The research protocol was approved by the Research Ethics Committee involving Human Subjects of the local University, under approval number 342.105, dated April 2024. All procedures complied with institutional and national guidelines for research involving human biological materials.

### Sample size calculation

Sample size calculation was performed using G*Power software (Heinrich Heine University Düsseldorf, Düsseldorf, Germany). The primary outcome was eroded dentin surface loss (dSL-eroded), based on data from a previous in vitro dentin erosion study with a comparable experimental design [11]. Considering the most conservative difference between active treatment groups in the present model—*Euterpe oleracea* (0.84 ± 0.06) versus *Byrsonima crassifolia* (0.98 ± 0.25) for dSL-eroded—and adopting a significance level of α = 0.05 and a statistical power of 80%, the minimum required sample size was estimated at 30 specimens per group.

### Saliva collection

Twenty healthy volunteers (8 males and 12 females), aged 18 to 30 years, participated in this study for whole saliva collection. Individuals were excluded if they were smokers, pregnant, under continuous medication, or presented with any diagnosed systemic disorder. Additional exclusion criteria related to oral health included the presence of active carious lesions, erosive tooth wear, or periodontal disease. Only participants exhibiting normal salivary function were included, defined by stimulated salivary flow rates exceeding 1.0 mL min⁻¹ and unstimulated flow rates above 0.3 mL min⁻¹. All saliva samples were pooled, and the supernatants were obtained by centrifugation at 14,000 × g for 20 min at 4 °C. The clarified saliva was subsequently aliquoted and stored at − 80 °C until experimental use [22].

### Dentin sample preparation

Human third molars were kept immersed in a 4% formaldehyde solution (pH 7.0) at room temperature until the moment of use. From these teeth, 180 dentin specimens (3 × 3 mm) were obtained from the cervical region. The teeth were sectioned at the cementoenamel junction with a precision cutting unit (Minitom, Struers S.A.S., Champigny-sur-Marne, France) operating at low speed and equipped with a diamond-coated dual-sided blade.

To standardize the dimensions, the cutting system was further adjusted with two parallel diamond disks (Extec Corp., Enfield, Lake Bluff, IL, USA) separated by a 2 mm spacer. All cutting procedures were performed under continuous irrigation with deionized water to minimize heat generation and prevent structural damage.

Following sectioning, the dentin blocks were sequentially abraded using silicon carbide abrasive papers of #600 and #1200 grit (Buehler, Lake Bluff, IL, USA) under running water, resulting in flat and polished surfaces. The prepared specimens were sonicated in deionized water for 5 min to remove debris and subsequently immersed in deionized water within a sealed container to maintain moisture. The humid chamber was stored inside a refrigerator (Electrolux^®^, Curitiba, PR, Brazil) at approximately 4 °C to prevent dehydration of the dentin substrates [22].

### Specimen selection and baseline microhardness evaluation

A total of 230 dentin blocks were initially screened to determine baseline surface microhardness. Measurements were obtained using a Knoop microhardness tester (Surftest, Mitutoyo South America, São Paulo, Brazil) with a static load of 50 g applied for 5 s [11]. Five indentations were made in the central area of each specimen, spaced 100 μm apart to avoid overlapping impressions.

Normality of the resulting data was verified using the Shapiro–Wilk test, performed with SPSS software (version 13.0; SPSS Inc., Tulsa, OK, USA). Fifty specimens exhibiting outlier hardness values were excluded from the analysis. The remaining 180 dentin blocks were sequentially numbered and randomly allocated into six experimental groups (*n* = 30 per group).

Statistical analysis confirmed that the mean baseline microhardness did not differ significantly among groups (one-way analysis of variance, ANOVA; α = 0.05).

### Induction of initial erosive lesion

The initial demineralization procedure was performed by immersing each dentin specimen in 3 mL of a citric acid solution (pH 3.6) for 10 min, without agitation, following a previously validated protocol [11, 23]. The procedure was conducted using 24-well acrylic plates, with one specimen placed per well to ensure uniform exposure.

After immersion, the samples were gently rinsed with distilled water for 10 s using a calibrated micropipette and then carefully blotted dry with absorbent paper. To delimit the area to be subsequently exposed, one half of each eroded surface was covered with unplasticized polyvinyl chloride (UPVC) adhesive tape, leaving a standardized 3 × 1.5 mm window of exposed dentin [11, 23].

### Erosive–abrasive cycling and experimental design

After allocation into the experimental groups (*n* = 30), the specimens were assigned to different outcome assessments. Fifteen specimens were used for profilometric analysis (dSL and dCol), and the remaining fifteen were used for calcium release (CaR) measurements, ensuring that each analysis was performed on independent specimens.

The dentin specimens were randomly allocated into six experimental groups (*n* = 30 per group): G1 – negative control, treated with Milli-Q water; G2 – experimental solution containing the seed extract of *Euterpe oleracea* (açaí); G3 – experimental solution containing the seed extract of *Bactris gasipaes* (peach palm); G4 – experimental solution containing the seed extract of *Paullinia cupana* (guaraná); G5 – experimental solution containing the seed extract of *Byrsonima crassifolia* (murici); G6 – positive control, treated with a commercial anti-erosive toothpaste containing 0.454% stannous fluoride (SnF₂; ~1100 ppm F⁻) - Oral-B Pro-Expert / Pro-Repair Anti-Erosion (Procter & Gamble, UK/EU).

At the beginning of the erosive–abrasive cycling, the treatment solutions were applied to the specimens according to their respective experimental groups. For this purpose, 1 µL of each solution was carefully dispensed onto the surface of each specimen using a single-channel pipette (Lambda^®^ Plus, Corning^®^, Sigma-Aldrich, Darmstadt, Germany), while the specimens were positioned in 24-well plates. Each solution remained in contact with the dentin surface for 10 min, following a protocol adapted from Alencar et al. [23]

The positive control group was treated with a 1:3 slurry prepared from the commercial anti-erosive toothpaste Oral-B Pro-Expert / Pro-Repair Anti-Erosion. Following treatment, the specimens were gently blotted dry with soft absorbent paper and immersed in 1 mL of freshly thawed human saliva containing protease inhibitors for 60 min at 37 °C to allow the initial adsorption of salivary proteins and the formation of an acquired pellicle on the dentin surface.

From day 1 to day 7, the specimens were subjected to daily erosive–abrasive challenges. Three times per day, the erosive challenges were performed by immersing the specimens in 1% citric acid solution (pH 3.6, 30 mL per specimen) without agitation for 90 s at 25 °C, followed by abrasion using an automatic toothbrushing machine (Odeme^®^, São Carlos, Brazil). Ultra-soft toothbrushes (Curaprox^®^ 5460, Kriens, Switzerland; one brush per specimen) were used without dentifrice at 37 °C according to the established abrasive protocol. After brushing, the specimens were rinsed with deionized water for 5 s and immersed in human saliva (pH 6.8, 30 mL per specimen) without agitation, following a protocol adapted from Alencar et al. [23]

### Dentin surface loss

The sample surfaces were gently cleaned with soft paper prior to profilometric analysis to reduce the presence of the acquired pellicle. Eroded dentin surface loss (dSL-eroded) was determined by calculating the difference between the mean height of the central exposed area (approximately 3 × 1.5 mm) and the mean height of the two adjacent reference areas, using dedicated software (FRT Mark III, FRT The Art of Metrology, Germany). Initial surface profiles, corresponding to baseline curvature, were subtracted from the final measurements to compensate for specimen-specific surface curvature (*n* = 15).

Subsequently, the dentin specimens were re-covered with adhesive tape positioned on the same reference areas as previously. The demineralized organic matrix was then removed by immersing each specimen in a saline solution containing type VII collagenase from *Clostridium histolyticum* (100 U/mL; C0773, Sigma-Aldrich) for 96 h at 37 °C under constant agitation (70 rpm). After collagenase treatment, the adhesive tapes were removed, and the same surface region was re-evaluated using the optical profilometer (MicroProf 100, FRT The Art of Metrology, Germany). Total dentin surface loss (dSL-total) was then determined. The amount of degraded collagen (dCol) was calculated as the difference between dSL-eroded and dSL-total [11, 24].

Scanning electron microscopy (SEM) images were obtained using a scanning electron microscope (MIRA3, TESCAN, Czech Republic) operating at an accelerating voltage of 5.0 kV in secondary electron mode [25]. For each group, representative micrographs were selected from the specimens to illustrate the surface morphology and the general appearance of dentin after treatment. The images were used for qualitative assessment only and serve as illustrative support for the findings, highlighting differences in dentinal tubule exposure and the presence of surface deposits among the groups.

### Calcium released to the citric acid (CaR)

To quantify dentin mineral loss, the calcium released to the citric acid after the erosive cycles was measured. For the total calcium release, 1 mL of citric acid after each erosive cycle was mixed per specimen and analyzed with an atomic absorption spectrometer (Aanalyst 400, Perkin-Elmer Analytical Instruments). The measured amount of calcium was normalized to the exposed dentin surface area (*n* = 15). The total amount of calcium release (CaR) per specimen was estimated considering the erosive cycles.

### Statistical analysis

Normality and homogeneity of variance were assessed using the Shapiro–Wilk and Levene tests, respectively. One-way ANOVA was performed to compare the experimental groups for each response variable: total dentin surface loss (dSL), degraded collagen (dCol), and calcium release (CaR). When significant differences were detected, Tukey’s post hoc test was applied. Total polyphenol content and antioxidant capacity were expressed as mean values obtained from repeated analytical measurements and compared using one-way ANOVA followed by Tukey’s test. Additionally, exploratory linear regression analyses were performed to investigate the association between total polyphenol content and dentin outcomes (dSL, dCol, and CaR). Due to the limited number of experimental extract groups and the potential interdependence among antioxidant parameters, total polyphenol content was selected as the primary predictor variable. Regression analyses were restricted to the experimental extract groups (G2–G5) and interpreted with caution. The significance level was set at 5% (α = 0.05). All statistical analyses were performed using SPSS software (IBM SPSS Statistics, version 26.0; IBM Corp., Armonk, NY, USA).

## Results

### Characterization of fruit-derived polyphenolic solutions

The pH values of the experimental solutions were close to neutral, ranging from 6.73 to 7.12. *Paullinia cupana* (guaraná) presented a pH of 7.02, *Euterpe oleracea* (açaí) 6.84, *Byrsonima crassifolia* (murici) 7.12, and *Bactris gasipaes* (peach palm) 6.73.

The total polyphenol content (TPC) and antioxidant capacity of the aqueous seed extracts are presented in Table 1. Among the fruit species tested, Paullinia cupana (guaraná) exhibited the highest TPC value, followed by Euterpe oleracea (açaí), Byrsonima crassifolia (murici), and Bactris gasipaes (peach palm).

**Table 1:** Polyphenol content and antioxidant capacity of aqueous extracts from tropical fruit seeds

Group - Fruits	Extractable polyphenols (mg GAE/100 g)	DPPH• EC50 (g/g DPPH)^b^	ABTS•^+^ µmol trolox/g
(G2) - *Euterpe oleracea*	1554.2 ± 54.6	663.4 ± 19.3	91.5 ± 7.4
(G3) *Bactris gasipaes*	82.5 ± 14.8	7482.2 ± 107.2	17.2 ± 1.5
(G4) - *Paullinia cupana*	2694.1 ± 39.7	466.9 ± 12.8	164.4 ± 14.9
(G5) *Byrsonima crassifolia*	184.3 ± 13.5	1709.7 ± 97.4	53.1 ± 2.9

^B^Concentration of antioxidant required to reduce the initial amount of free radicals by 50%

Consistently, the P. cupana extract showed the lowest DPPH EC₅₀ value and the highest ABTS radical-scavenging activity, indicating superior antioxidant capacity. E. oleracea displayed intermediate performance, whereas B. crassifolia and B. gasipaes showed lower antioxidant responses, with markedly higher DPPH EC₅₀ values. Overall, the extracts followed the descending order of antioxidant potential: P. cupana (guaraná) > E. oleracea (açaí) > B. crassifolia (murici) > B. gasipaes (peach palm).

Following the classification proposed by Vasco et al., [26] who analyzed tropical fruits for their polyphenol content, the samples were categorized into three groups based on fresh weight: low (< 100 mg GAE/100 g), medium (100–500 mg GAE/100 g), and high (> 500 mg GAE/100 g).

### Dentin surface loss

The 3D profilometry analysis revealed significant differences in surface loss and collagen degradation among the tested groups (Table 2). The greatest dentin surface loss (dSL-eroded) and collagen degradation (dCol) were observed in the negative control group treated with Milli-Q water. Conversely, the lowest values were recorded for the group treated with *Paullinia cupana* (guaraná) seed extract, indicating the most pronounced protective effect against erosive–abrasive challenges (*p* = 0.029).

**Table 2:** Mean (M) and standard deviation (±SD) of 3D profilometry values after erosive–abrasive cycling (dSL-eroded)

Group	The total dentin surface loss (dSL-eroded)	Dentin degraded collagen (dCol)
G1	5.13 (± 0.72)^A^	2.52 (± 0.64)^A^
G2	0.84 (± 0.06)^B^	0.23 (± 0.07)^B^
G3	3.61 (± 0.85)^A^	1.79 (± 0.12)^A^
G4	0.39 (± 0.03)^C^	0.18 (± 0.03)^C^
G5	0.98 (± 0.25)^B^	0.37 (± 0.03)^B^
G6	1.53 (± 0.63)^D^	0.75 (± 0.11)^D^

Note: Capital letters represent statistically significant intergroup difference (*p* < 0.05). Groups description - (G1 – negative control, treated with Milli-Q water; G2 – experimental solution containing the seed extract of *Euterpe oleracea* (açaí); G3 – experimental solution containing the seed extract of *Bactris gasipaes* (peach palm); G4 – experimental solution containing the seed extract of *Paullinia cupana* (guaraná); G5 – experimental solution containing the seed extract of *Byrsonima crassifolia* (murici); G6 – positive control, treated with a commercial anti-erosive toothpaste containing 0.454% stannous fluoride (SnF₂; ~1100 ppm F⁻) - Oral-B Pro-Expert / Pro-Repair Anti-Erosion (Procter & Gamble, UK/EU)

Intermediate performance was observed for the groups treated with *Euterpe oleracea* (açaí) and *Byrsonima crassifolia* (murici), which did not differ significantly from each other (*p* > 0.05). The *Bactris gasipaes* (peach palm) extract (G3) showed higher surface loss and collagen degradation compared with the other fruit seed extracts, although still significantly lower than the untreated control (*p* = 0.037).

Additionally, exploratory linear regression analyses suggested a negative association between total polyphenol content and dentin outcomes. Exploratory analyses suggested an inverse relationship between total polyphenol content and dSL-eroded (β = −0.0016, R² = 0.96, *p* = 0.02), as well as dCol (β = −0.0009, R² = 0.94, *p* = 0.03), indicating that extracts with higher polyphenol levels were associated with reduced tissue breakdown.

**Fig. 1:**
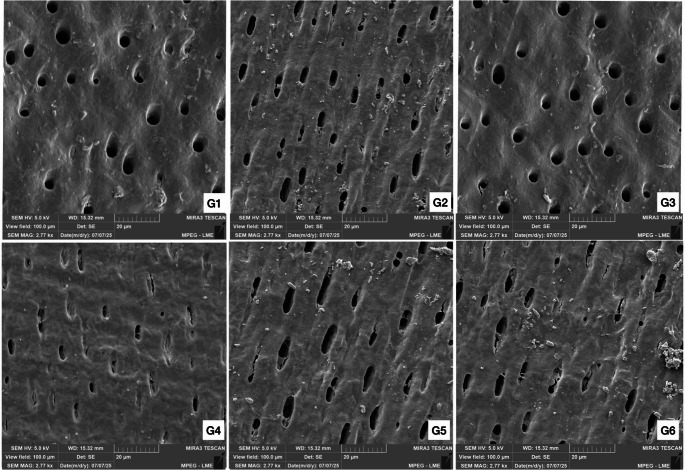
Representative scanning electron microscopy (SEM) micrographs of dentin surfaces after erosive–abrasive cycling. G1: Milli-Q water (negative control); G2: Euterpe oleracea; G3: Bactris gasipaes; G4: Paullinia cupana; G5: Byrsonima crassifolia; and G6: 0.454% stannous fluoride (SnF₂). Qualitative differences in surface morphology and dentinal tubule exposure were observed among the experimental groups. Original magnification: 2.77 k×; scale bar = 20 μm

Figure 1 presents representative SEM micrographs illustrating the surface morphology of dentin specimens across the experimental groups, highlighting differences in dentinal tubule exposure and surface deposits.

### Calcium released to the citric acid (CaR)

Significant differences in calcium release were observed among the experimental groups (*p* < 0.05) (Table 3). The negative control exhibited the highest mineral loss, whereas all experimental extracts significantly reduced calcium release compared with the untreated group (*p* = 0.029). The *Paullinia cupana* (guaraná) extract demonstrated the most pronounced protective effect (*p* = 0.047), followed by *Euterpe oleracea* (açaí) and *Byrsonima crassifolia* (murici), which showed similar performance. In contrast, the *Bactris gasipaes* (peach palm) extract exhibited more limited protection, while the stannous fluoride group presented intermediate values between the experimental extracts and the negative control.

**Table 3:** Mean (M) and standard deviation (± SD) of values total calcium released to the citric acid after erosive–abrasive cycling (CaR)

Group	(CaR) (*nmol Ca/mm*^*2*^ *dentin*)
G1	79.33 (± 11.01)^A^
G2	25.75 (± 8.14)^B^
G3	63.89 (± 10.27)^A^
G4	11.23 (± 5.70)^C^
G5	28.95 (± 6.09)^B^
G6	43.07 (± 10.82)^D^

Note: Capital letters represent statistically significant intergroup difference (*p* < 0.05). Groups description - (G1 – negative control, treated with Milli-Q water; G2 – experimental solution containing the seed extract of *Euterpe oleracea* (açaí); G3 – experimental solution containing the seed extract of *Bactris gasipaes* (peach palm); G4 – experimental solution containing the seed extract of *Paullinia cupana* (guaraná); G5 – experimental solution containing the seed extract of *Byrsonima crassifolia* (murici); G6 – positive control, treated with a commercial anti-erosive toothpaste containing 0.454% stannous fluoride (SnF₂; ~1100 ppm F⁻) - Oral-B Pro-Expert / Pro-Repair Anti-Erosion (Procter & Gamble, UK/EU)

Exploratory linear regression analysis indicated a negative association between total polyphenol content and CaR (β = −0.018, R² = 0.97, *p* = 0.01), suggesting that extracts with higher polyphenol levels tended to reduce mineral loss.

## Discussion

This study evaluated the protective potential of experimental solutions obtained from the seeds of four tropical fruits — *Paullinia cupana* (guaraná), *Euterpe oleracea* (açaí), *Byrsonima crassifolia* (murici), and *Bactris gasipaes* (peach palm) — on the organic–inorganic interface of eroded dentin. The results demonstrated significant differences among the evaluated treatments, indicating distinct anti-erosive behaviors under erosive–abrasive conditions. These effects may be related to differences in the chemical composition of the extracts, including their polyphenolic content and antioxidant capacity, although the contribution of individual components cannot be isolated within the present experimental design.

Currently, there is no gold-standard treatment capable of fully preventing the progression of erosive tooth wear, particularly in dentin, where the organic matrix plays a crucial role in structural integrity [17]. Therefore, the development of natural, biocompatible, and bioactive strategies represents a relevant and innovative approach in preventive dentistry [27, 28]. In this context, the present findings provide preliminary evidence supporting the potential of plant-derived extracts as multifunctional agents and may contribute to future translational applications, including the sustainable use of Amazonian biodiversity in the design of dentin-protective formulations.

The null hypothesis was rejected, as significant differences were observed among the evaluated treatments for dentin surface loss, collagen degradation, and calcium release. Among the tested extracts, *P. cupana* (guaraná) exhibited the most pronounced protective effect, which was consistent with its higher total polyphenol content and antioxidant capacity (lowest DPPH EC₅₀ and highest ABTS activity). Although these findings suggest a relationship between chemical composition and protective performance, a direct causal effect cannot be established. Exploratory regression analyses further indicated a negative association between total polyphenol content and dentin outcomes, suggesting that extracts richer in polyphenols tended to be associated with improved protective behavior. Despite the limited number of experimental extract groups, the consistency of this trend across multiple outcomes — including dentin surface loss, collagen degradation, and calcium release — supports the biological plausibility of this association. Nevertheless, these findings should be interpreted with caution due to the exploratory nature of the analysis and the complex composition of the tested solutions. Further studies using more controlled experimental designs and larger datasets are needed to clarify the extent to which polyphenol content contributes to the observed protective effects.

The pronounced protective effect observed for *P. cupana* (guaraná) may be related to its well-documented antioxidant profile, which has been attributed to the presence of catechins, epicatechins, and condensed tannins capable of interacting with collagen fibrils and contributing to the stabilization of the organic matrix [29, 30]. However, to the best of the authors’ knowledge, no previous study has investigated the effects of guaraná — either from the fruit or its seeds — on eroded dentin or other mineralized dental tissues. Thus, the present findings provide novel experimental evidence supporting the dentin-protective potential of *P. cupana*–derived compounds. A similar trend, although less pronounced, was observed for *E. oleracea* (açaí), which exhibited a moderate but significant protective effect, consistent with previous reports describing its high antioxidant capacity [31, 32]. The performance of *B. crassifolia* (murici) was also in agreement with earlier findings demonstrating the presence of flavonoids and tannins with reducing and chelating activity [33]. In contrast, *B. gasipaes* (peach palm) showed the lowest protective performance among the tested extracts, which may be associated with its lower polyphenol content and distinct chemical composition.

In fact, *B. gasipaes* seed extracts are known to be predominantly composed of lipophilic constituents, such as saturated fatty acids, tocopherols, and carotenoids [34]. Neves et al. [34] reported that the oil extracted from peach palm seeds is particularly rich in lauric acid (49.82%) and myristic acid (21.53%), which contribute to its oxidative stability and hydrophobic properties. Although these characteristics support potential industrial applications, such compounds may not effectively interact with the dentin substrate or promote the formation of a stable protective surface layer. This compositional profile may help explain the reduced dentin-protective performance observed for *B. gasipaes* in the present study. Unlike polyphenol-rich extracts, these lipophilic molecules appear to have limited ability to modulate the organic matrix or contribute to dentinal tubule occlusion.

These chemical differences may help explain the distinct anti-erosive behaviors observed among the tested extracts. Low-molecular-weight polyphenols, such as catechins and proanthocyanidins—particularly abundant in guaraná and açaí—have been reported to interact with collagen fibrils through hydrogen bonding and potential covalent interactions, contributing to the biomodification of the dentin matrix. Such interactions may promote the formation of exogenous cross-links within and between collagen molecules, enhancing fibrillar cohesion, reducing susceptibility to enzymatic degradation, and improving the mechanical and chemical stability of the organic matrix [35].

In addition, the reduced erosive wear observed in the present study may not be exclusively related to protection against mineral loss. The formation of exogenous cross-links within the collagen matrix may increase its resistance to deformation and degradation, thereby contributing to greater stability of the dentin substrate under erosive–abrasive conditions. This interpretation is consistent with previous studies demonstrating that biomodification of the dentin organic matrix can influence its behavior during erosive challenges [12]. Therefore, the reduced dentin surface loss observed in some groups may reflect a combined effect of erosion prevention and collagen biomodification. These effects are often attributed to the high density of hydroxyl groups present in polyphenols, which enables multivalent interactions with amino acid residues in collagen, particularly hydroxyproline and lysine. As a result, the structural framework of demineralized dentin may be reinforced, potentially limiting water diffusion and the activity of proteolytic enzymes, thereby reducing collagen solubilization and degradation over time [35]. However, these mechanisms were not directly assessed in the present study and should therefore be interpreted as plausible explanations rather than definitive evidence.

In addition, the antioxidant and metal-chelating properties of these compounds may contribute to the modulation of matrix metalloproteinases (MMPs) and cysteine cathepsins, enzymes involved in collagen degradation under acidic and post-erosive conditions [36]. By helping to maintain redox balance and stabilize the collagen network, these compounds may support the preservation of the organic scaffold of dentin and favor the re-precipitation of calcium and phosphate ions on the exposed matrix, potentially resulting in a more acid-resistant substrate. Consistent with this rationale, previous studies have demonstrated the anti-erosive potential of antioxidant-based strategies. Manzoli et al. [11] showed that a gel containing resveratrol and sodium fluoride significantly reduced dentin surface loss and enhanced the protective effect of the acquired salivary pellicle. These findings support the concept that antioxidant compounds may act synergistically with fluoride in protecting the dentin surface and preserving the organic–inorganic interface.

In the present study, the improved performance observed for *P. cupana* and *E. oleracea* may be associated with these combined mechanisms, including antioxidant activity and potential interactions with the dentin matrix. However, given the complex composition of the extracts, it is not possible to attribute the observed effects exclusively to polyphenols or to a single mechanism of action. Consistent with these findings, the null hypothesis was rejected, as significant differences were observed among the evaluated treatments for all dentin outcomes. The marked reduction in calcium release in the groups treated with *P. cupana*, *E. oleracea*, and *B. crassifolia* suggests lower mineral dissolution and the possible formation of more stable organic–inorganic interactions at the dentin surface, in agreement with mechanisms previously described for antioxidant-based systems [17].

This effect may be related to the ability of polyphenols to interact with calcium and phosphate ions and potentially favor their re-adsorption onto the demineralized substrate, which could contribute to remineralization processes and reduce the solubility of hydroxyapatite crystals [36–40]. Moreover, the interaction of phenolic hydroxyl groups with exposed collagen fibrils may contribute to the formation of a protective organic–inorganic interface, potentially enhancing mechanical stability and resistance to acid challenges [40–42]. These observations are consistent with the hypothesis that plant-derived antioxidants may act not only as radical scavengers but also as modulators of the dentin matrix and ionic environment, contributing to reduced surface demineralization [11, 22, 42]. However, it should be emphasized that such mechanisms were not directly evaluated in the present study and should therefore be interpreted as plausible explanations rather than definitive evidence.

In addition, the present findings are in agreement with recent studies demonstrating the potential dual protective role of plant-derived antioxidants on dentin. Niemeyer et al. [42] reported that certain botanical extracts may act simultaneously on the dentin substrate and the acquired salivary pellicle, reducing demineralization and enhancing pellicle-mediated protection against acid challenges. Similarly, Niemeyer et al. [24] observed that the association of plant extracts with fluoride may produce additive effects, reinforcing both organic and inorganic components of dentin and resulting in improved anti-erosive performance compared with fluoride alone. Taken together, these findings support the concept that bioactive phytochemicals, such as polyphenols, may contribute to the stabilization of the dentin collagen matrix while also influencing the composition and function of the acquired pellicle, potentially leading to a more acid-resistant and mechanically stable surface.

In contrast, the stannous fluoride formulation is known to reduce erosion primarily through the formation of a tin-rich protective layer on the mineral surface, thereby enhancing resistance to acid challenges and reducing permeability [43]. However, this mechanism is mainly associated with the inorganic phase of dentin and may present reduced effectiveness under conditions in which the organic matrix is exposed and plays a key role in substrate integrity [44], as in the erosive–abrasive model employed in this study. In addition, differences in the formulation vehicle may have influenced the observed performance. The stannous fluoride was applied as a dentifrice, which typically presents higher viscosity and may limit its penetration and interaction with the dentin substrate compared with low-viscosity solutions. This could reduce the availability of active ions at the substrate interface under the experimental conditions used. By comparison, the plant-derived extracts were applied as solutions, which may facilitate greater contact with the exposed dentin matrix and potentially enhance their interaction with both organic and inorganic components. Nevertheless, these mechanisms were not directly assessed, and the observed differences should be interpreted within the context of the experimental design.

Scanning electron microscopy images provided qualitative support for the observed trends, revealing distinct surface morphologies among treatments. Specimens treated with *P. cupana* (guaraná) and *E. oleracea* (açaí) exhibited relatively smoother and more compact surfaces, with reduced apparent dentinal tubule exposure, suggesting a potential protective effect at the surface level. These observations are in agreement with previous reports indicating that reduced tubule exposure and surface densification may reflect the presence of protective surface deposits [11, 30]. In contrast, the untreated control and *B. gasipaes* groups showed more pronounced tubule exposure and surface irregularities, features commonly associated with greater mineral loss. It should be noted, however, that SEM analysis was performed qualitatively and on selected specimens, and therefore should be interpreted as illustrative rather than as definitive evidence.

The formation of a more compact surface layer in specimens treated with polyphenol-rich extracts may be explained by interactions between these compounds and the dentin substrate. Polyphenols have been reported to interact with calcium and phosphate ions, potentially contributing to the formation of weakly mineralized surface complexes that could partially occlude dentinal tubules [42]. In addition, the adsorption of polyphenolic molecules onto exposed collagen fibrils may influence surface properties, such as wettability and permeability, while contributing to the stabilization of the organic network through hydrogen bonding and possible cross-linking. The hypothesis of calcium–tannin complexation may also be consistent with these observations; however, this mechanism remains speculative, as the specific tannin content of the extracts was not quantified in the present study. Nevertheless, the combined findings of reduced calcium release and altered surface morphology suggest that some degree of interaction between polyphenols and the dentin substrate may have occurred, potentially contributing to increased resistance to acid challenges.

The stannous fluoride formulation exhibited a protective effect primarily associated with inorganic tin deposition, acting mainly on the mineral component of dentin without directly stabilizing the organic matrix [43]. In contrast, the performance observed for *P. cupana* and *E. oleracea* may be related to a multifactorial behavior, involving antioxidant activity and potential interactions with both organic and inorganic components of dentin. However, given the complex composition of the extracts, these effects cannot be attributed to a single mechanism. Despite the promising outcomes of this in vitro study, several limitations should be considered. The experimental conditions do not fully reproduce the complex biological and mechanical environment of the oral cavity, where factors such as salivary flow, enzymatic activity, and biofilm interactions play a critical role in the progression of erosive tooth wear. In addition, the study did not include a detailed phytochemical characterization of each extract, such as the quantitative determination of tannins or specific polyphenol subclasses that may be responsible for the observed effects.

Further studies are therefore warranted to isolate and identify the active compounds present in guaraná and açaí seed extracts, as well as to evaluate their cytocompatibility and long-term performance under dynamic oral conditions. Future investigations using in situ and in vivo models, along with the incorporation of these extracts into delivery systems such as gels, rinses, or restorative materials, may provide valuable insights for the development of sustainable, plant-based strategies for dentin protection.

## Conclusion

Within the limitations of this in vitro study, polyphenol-rich solutions derived from tropical fruit seeds, particularly *Paullinia cupana* (guaraná) and *Euterpe oleracea* (açaí), were associated with reduced dentin surface loss, collagen degradation, and calcium release under erosive–abrasive conditions. Exploratory regression analyses further suggested an inverse association between total polyphenol content and dentin outcomes, supporting the biological plausibility of a protective effect. However, due to the complex composition of the extracts, the contribution of individual phytochemical constituents could not be determined. Therefore, the underlying mechanisms should be further clarified in future studies.

## Data Availability

The datasets generated and/or analyzed during the current study are available from the corresponding author on reasonable request.
